# *Nitrosospira* sp. Govern Nitrous Oxide Emissions in a Tropical Soil Amended With Residues of Bioenergy Crop

**DOI:** 10.3389/fmicb.2018.00674

**Published:** 2018-04-10

**Authors:** Késia S. Lourenço, Noriko A. Cassman, Agata S. Pijl, Johannes A. van Veen, Heitor Cantarella, Eiko E. Kuramae

**Affiliations:** ^1^Department of Microbial Ecology, Netherlands Institute of Ecology, Wageningen, Netherlands; ^2^Soils and Environmental Resources Center, Agronomic Institute of Campinas, Campinas, Brazil; ^3^Institute of Biology Leiden, Leiden University, Leiden, Netherlands

**Keywords:** nitrogen and carbon biochemical cycles, recycling, vinasse, quantitative real time PCR, *amo*A gene, sugarcane

## Abstract

Organic vinasse, a residue produced during bioethanol production, increases nitrous oxide (N_2_O) emissions when applied with inorganic nitrogen (N) fertilizer in soil. The present study investigated the role of the ammonia-oxidizing bacteria (AOB) community on the N_2_O emissions in soils amended with organic vinasse (CV: concentrated and V: non-concentrated) plus inorganic N fertilizer. Soil samples and N_2_O emissions were evaluated at 11, 19, and 45 days after fertilizer application, and the bacterial and archaea gene (*amo*A) encoding the ammonia monooxygenase enzyme, bacterial denitrifier (*nir*K, *nir*S, and *nos*Z) genes and total bacteria were quantified by real time PCR. We also employed a deep *amo*A amplicon sequencing approach to evaluate the effect of treatment on the community structure and diversity of the soil AOB community. Both vinasse types applied with inorganic N application increased the total N_2_O emissions and the abundance of AOB. *Nitrosospira* sp. was the dominant AOB in the soil and was correlated with N_2_O emissions. However, the diversity and the community structure of AOB did not change with vinasse and inorganic N fertilizer amendment. The results highlight the importance of residues and fertilizer management in sustainable agriculture and can be used as a reference and an input tool to determine good management practices for organic fertilization.

## Introduction

Brazil is the world’s largest producer of sugarcane, with about 685 million tons of sugarcane produced from an area of 9 million hectares in 2016/2017 (CONAB, 2017). Roughly 53% of sugarcane production is directed toward bioethanol production, which is considered a sustainable biofuel (CONAB, 2017). Studies conducted by Macedo et al. (2008) and Seabra et al. (2011) indicated that ethanol production from sugarcane emits about 80% less greenhouse (GHG) gasses than the production of fossil fuels. These benefits are reduced during the practice of recycling sugarcane straw and bioethanol production residues (Galdos et al., 2010; De Figueiredo and La Scala, 2011; Carmo et al., 2013; Pitombo et al., 2015; Siqueira Neto et al., 2016; Soares et al., 2016). For each liter of ethanol produced, 10–15 liters of the liquid waste, called vinasse, are generated, which totals roughly 360 billion liters of vinasse per year. Vinasse is the major residue generated during the production of ethanol from sugarcane. Vinasse is rich in organic content, carbon (10,000–20,000 mg C L^–1^), nitrogen (357 mg N L^–1^), and especially potassium (2056 mg L^–1^) (Elia-Neto and Nakahodo, 1995; Macedo et al., 2008; Christofoletti et al., 2013; Fuess and Garcia, 2014). To recycle these nutrients, vinasse is directly applied on sugarcane fields as a fertilizer. Recently the use of concentrated vinasse fertilizer, made by reducing the water volume of vinasse, has become popular due to the overall reduced cost associated with transporting the fertilizer to the field. Thus, both types concentrated and non-concentrated vinasse are currently used as organic fertilizer (Rodrigues Reis and Hu, 2017). However, when vinasse is applied with N fertilizer in the soil, high nitrous oxide (N_2_O) emission occur (Carmo et al., 2013; Paredes et al., 2014, 2015; Pitombo et al., 2015).

Nitrous oxide is one of the molecules of the nitrogen (N) cycle with major environmental and ecological impacts. N_2_O is both an ozone-depletion substance (Ravishankara et al., 2009) and a GHG with global warming potential 298 times higher than carbon dioxide (CO_2_) (IPCC, 2013). Agricultural soils account for an estimated 65% of global N_2_O emissions (IPCC, 2013). N_2_O is produced in soil via biotic and abiotic processes. Chemodenitrification is an abiotic process and it is carried out in low pH (<4.5) by the chemical decomposition of hydroxylamine (NH_2_OH), nitroxyl hydride (HNO) or NO_2_^–^ with organic and inorganic compounds. Biotic N_2_O production processes are widely distributed over the soil microbiota and have been observed in more than 60 bacterial and archaeal genera (Philippot et al., 2007; Canfield et al., 2010; Nelson et al., 2016). N_2_O can be emitted as a byproduct of nitrification or denitrification, which are the main biotic processes contributing to N_2_O emissions in soil (Goreau et al., 1980; Wrage et al., 2001; Bakken and Frostegård, 2017). Denitrification is widely responsible for soil N_2_O emissions at high water contents while nitrification has often been assumed to be the principal source of N_2_O in soil under aerobic conditions (Mathieu et al., 2006; Soares et al., 2016).

Nitrification is the aerobic oxidation of ammonia (NH_3_) to nitrate (NO_3_^–^), which occurs in two phases mediated mainly by autotrophic microorganisms. In the first phase, ammonia-oxidizing bacteria (AOB) or archaea (AOA) oxidize NH_3_ to nitrite (NO_2_^–^); in the second phase, nitrite-oxidizing bacteria (NOB) oxidize NO_2_^–^ to NO_3_^–^. The ammonia oxidation phase (NH_3_ → NH_2_OH/HNO → NO_2_^–^) is catalyzed by the ammonia monooxygenase enzyme encoded by the *amo*A gene, which is carried by β- or γ-proteobacteria (AOB) and the newly described *Thaumarchaeota* phylum (AOA). The *nxr*B gene encodes the enzyme nitrite oxidoreductase and regulates the second phase of nitrification. The N_2_O production by AOB is the result of incomplete oxidation of NH_2_OH to either nitroxyl (HNO) or NO (Smith and Hein, 1960; Hu et al., 2015) which occurs under aerobic conditions. The second N_2_O^–^ yielding route related to nitrifiers is termed nitrifier denitrification and occurs under both high and low oxygen concentrations. In this process, AOB possess machinery that reduces NO_2_^–^ to N_2_O via a nitric oxide (NO) intermediate (Ritchie and Nicholas, 1972; Shaw et al., 2006). Shi et al. (2017) showed that nitrifier denitrification is an important pathway leading to high N_2_O production (10.5–54.5%) in alkaline cropping soils (pH 7.2); however, heterotrophic denitrification accounted for a the largest proportion of total N_2_O production (∼83%) in acid soils (pH 4.9). Recently, Caranto et al. (2016) demonstrated another direct enzymatic pathway from NH_2_OH to N_2_O under anaerobic conditions, which is mediated by cytochrome P460. Furthermore, nitrification can also occur during a single step as performed by bacteria of the genus *Nitrospira* sp. (Daims et al., 2015; van Kessel et al., 2015); however, it is not yet known if N_2_O emissions occur during this process.

Denitrification is a multistep reaction performed by a variety of bacteria and fungi. During denitrification, oxidized mineral forms of N (NO_3_^–^ and NO_2_^–^) are reduced to the gaseous products NO, N_2_O, and N_2_ under oxygen-limited conditions (NO_3_^–^ → NO_2_^–^ → NO → N_2_O → N_2_). The sequential processes of bacterial denitrification are regulated by divergent reductases encoded by distinct functional genes: the *nar*G or *nap*A genes encode nitrate reductase, the *nirK* or *nirS* genes encode two entirely different types of nitrite reductase, the *nor*B encode nitric oxide reductase and the *nos*Z gene encodes N_2_O reductase (Philippot et al., 2007; Jones et al., 2013).

In a recent study conducted in sugarcane fields in Brazil, AOB rather than AOA or denitrifier bacteria were associated with N_2_O emissions (Soares et al., 2016), suggesting that nitrification is the dominant N_2_O-producing process in these soils. While it is known that using vinasse plus inorganic N fertilizers increases N_2_O emissions, there are no studies to date on the effects of these treatments on the AOB communities in these soils. Therefore, the aim of the current study was to evaluate the effects of vinasse plus inorganic nitrogen fertilization on the ammonia-oxidizing bacterial community abundance, structure, and diversity of the AOB in a tropical soil planted with sugarcane. We hypothesized that the abundance and community structure of the AOB would respond to organic and inorganic, i.e., vinasse and fertilization.

## Materials and Methods

### Experimental Setup and Soil Sampling

The field experiment was situated in Piracicaba, Brazil at APTA (Paulista Agency for Agribusiness Technology). The mean annual air temperature and precipitation of the region are 21°C and 1,390 mm, respectively. Precipitation and daily temperature measurements during the experiment were obtained from a meteorological station located nearby the experimental field. The soil was classified as Ferrasol (FAO, 2015) with pH of 5.0, organic matter of 21.1 g dm^–3^, P of 14.6 mg dm^–3^, K^+^ of 0.7 mmol_c_ dm^–3^, Ca^+2^ of 17.4 mmol_c_ dm^–3^, Mg^+2^ of 11.9 mmol_c_ dm^–3^, H^+^ + Al^+3^ of 34.9 mmol_c_ dm^–3^, CEC of 65.1 mmol_c_ dm^–3^, and soil bulk density of 1.49 g cm^–3^. The experiment was carried out in a field planted with the sugarcane variety RB86-7515. The sugarcane was mechanically harvested and the straw was left on top of the soil (16 Mg ha^–1^). The experiment was conducted in a randomized block design with three replicate blocks. The treatments were: (1) Control: plot without inorganic N fertilization or vinasse; (2) N: inorganic N fertilizer only; (3) CV+N: concentrated vinasse plus inorganic N fertilizer; and (4) V+N: non-concentrated vinasse plus inorganic N fertilizer.

The inorganic fertilizers and concentrated vinasse (CV) were surface-applied in a 0.2-m wide row, close to the plant (0.1 m) in agreement with common practices in commercial sugarcane production. The N fertilizer rate was 100 kg N ha^–1^ of ammonium nitrate (NH_4_NO_3_). Volumes of 1.0 × 10^5^ l ha^–1^ of non-concentrated vinasse (V) were sprayed over the experimental plots using a motorized pump fit with a flow regulator. This amount of V corresponded with recommended average application rates to sugarcane plantations in Sao Paulo. Concentrated vinasse was applied in fertilization rows at rate of 1.7 × 10^5^ l ha^–1^. CV was produced by concentrating vinasse by a factor of 5.8, which is the average of sugar mill vinasse concentration processes. The chemical characteristics of the vinasses are listed in Supplementary Table S1.

The experiment started on August 15, 2014 and 6 soil samplings per plot were carried out on three time points: 11, 19, and 45 days after inorganic N and vinasse applications. For each treatment, soil samples were collected from the 0–10 cm layer for measurements of moisture content, concentrations of NO_3_^–^-N and NH_4_^+^-N, and pH. Soil subsamples (30 g) were stored at –80°C for molecular analyses. In parallel, for each soil sample air and soil temperatures were measured. Soil temperatures were collected from the 0–10 cm layer with a digital thermometer. Soil moisture was determined gravimetrically by drying the soil at 105°C for 24 h and the water-filled pore space (WFPS) was calculated considering soil moisture and bulk density. Soil mineral N (NH_4_^+^-N, NO_3_^–^-N) was measured with a continuous flow analytical system (FIAlab-2500 System) (Kamphake et al., 1967; Krom, 1980).

### Nitrous Oxide Measurements

Fluxes of N_2_O were measured using PVC static chambers with 20 cm height and 30 cm diameter, according to the method described in Pitombo et al. (2015) and Soares et al. (2016). The gases were sampled with plastic syringes (60 mL) at three time intervals (1, 15, and 30 min) after the chambers were closed (Soares et al., 2016). The samples were transferred and stored in pre-evacuated 12 mL glass vials and analyzed in a gas chromatograph with an electron capture detector for N_2_O and with flame ionization detector for CO_2_ determination (model GC-2014, Shimadzu Co.). Gas and soil samples were collected in the morning between 7:00 and 12:00 AM GMT-3. Overall N_2_O flux was calculated by linear interpolation over the three sampling times.

### DNA Extraction and Real-Time PCR

Total soil DNA was extracted using the MoBio PowerSoil DNA Isolation Kit (MoBio, Solana Beach, CA, United States). Of each soil sample, 0.30 g was used for DNA extraction according to the manufacturer’s instructions. The quantity and quality of DNA were quantified and checked using a Qubit 2.0 Fluorometer (Life Technologies, Carlsbad, CA, United States), as well as visualized on 1% (w/v) agarose gel under UV light. The abundance of the *amo*A-AOB gene and total bacterial community was quantified by real-time PCR with a BIO-RAD CFX96 Touch^TM^ Real-Time PCR Detection System. Amplification of the *amo*A-AOB gene was performed in total volume of 12 μL, containing 6 μL SYBR Green Bioline SensiFAST SYBR^®^ No-ROX mix, 0.125 μL of each primer (10 pmol) and 4 μL of DNA (40 ng); the primer used was amoA1F (5′-GGGGTTTCTACTGGTGGT-3′) and amoA2R (5′-CCCCTCKGSAAAGCCTTCTTC-3′) (Rotthauwe et al., 1997). The thermal cycler conditions were 95°C-10 min; 40 times 95°C-10 s, 65°C-25 s; last, acquisition was done at 65°C. The qPCR amplicon products (491 bp) were checked by melting curve analysis and agarose gel electrophoresis. The efficiency of the *amo*A-AOB qPCR was 87% (*R*^2^ = 0.99). Assessment of the abundance of the total bacterial community was based on 16S rRNA gene qPCR was performed in total volume of 12 μL, containing 6 μL SYBR Green iQ^TM^ SYBR^®^ Green Supermix (Bio-Rad), 0.125 μL of each primer (10 pmol), 0.30 μL of bovine serum albumin (BSA), and 4 μL of DNA (5 ng); the primer sets were Eub338 (5′-ACTCCTACGGGAGGCAGCAG-3′) and Eub518 (5′-ATTACCGCGGCTGCTGG-3′) (Fierer et al., 2005). The thermal cycler conditions were 95°C-3 min; 40 times 95°C-30 s, 59°C-35 s; 72°C-20 s, and acquisition was done at 59°C. The qPCR amplicon products (200 bp) were checked by melting curve analysis and agarose gel electrophoresis. The efficiency of the 16S rDNA qPCR was 96% (*R*^2^ = 0.99). Plasmid DNA containing fragments of bacterial *amo*A and 16S rRNA genes were used as standards. Each qPCR run, in triplicate, included a DNA template, the standard positive control, and a negative control. The primers used for the archaea *amo*A and bacteria *nir*S, *nir*K, and *nos*Z and the, PCR conditions are described in the Supplementary Table S2.

### Sequencing of *amo*A Amplicons for Ammonia-Oxidizing Bacteria

Primer sets amoA-1F/amoA-2R (Rotthauwe et al., 1997) for AOB (same primers used in the qPCR) were used to amplify the *amo*A gene fragment for sequencing with Illumina MiSeq sequencing platform. The PCR was carried out in 20 μl reaction containing each 2 μl of deoxynucleoside triphosphate at a concentration of 2.0 mM, 0.25 μl of forward and reverse primers (10 pmol), 0.1 μl of FastStart Taq DNA Polymerase, 2 μl of MgCl_2_ buffer, and 0.5 μl of BSA (4 mg ml^–1^). Each reaction mix received 1 μl of genomic DNA as a template. The PCR conditions for the amplicons were: preheating at 95°C for 5 min, then 35 cycles (95°C for 30 s, 53°C for 30 s, and 72°C for 30 s), with a final extension at 72°C for 10 min. Triplicate reaction mixtures per sample were pooled together, purified with the Agarose Gel DNA purification kit (TaKaRa), and quantified using the NanoDrop ND-1000 spectrophotometer (NanoDrop Technologies, Montchanin, DE, United States). The bar-coded PCR products from all samples were normalized in equimolar amounts before sequencing. The amplicon library was prepared by adaptor ligation and PCR using the TruSeq Nano DNA Library Prep Kit (Illumina, CatFC-121-4001) according to the TruSeq nano protocol (Illumina, FC-121-4003). Paired-end MiSeq sequencing was carried out by BGI Inc. (China). The raw *amo*A sequence data are available at the European Nucleotide Archive (ENA)^1^ under the study accession number PRJEB25428.

### Clustering and Taxonomic Classification of *amo*A OTUs

The raw data of *amo*A sequences were preprocessed using MOTHUR v 1.3.3 (Schloss et al., 2009). Raw sequences were merged (make.contigs command), then trimmed and sorted simultaneously (trim.seqs). Sequences were filtered out if average read quality was less than 25, there were more than two N’s or if the read length was less than 150 bp; remaining sequences were filtered based on primer quality (≤ 2 errors), spacers (≤2 errors), and barcodes (≤1 error). Barcodes and primers were removed. Further, the sequences were processed using the UCLUST pipeline implemented in a Snakemake workflow which is available upon request (Edgar, 2010). In summary, the *amo*A-AOB sequences were truncated to 480 bp, clustered into 90% OTUs and singletons and chimeras were removed (Norton et al., 2002). An OTU table was created at the 90% cutoff level. The OTUs were checked by comparison to the March 17, 2014 KEGG database using UProC version 1.2.0 with the uproc-dna command (Meinicke, 2015); those OTUs that did not match the *pmo*A-*amo*A (Particulate methane monooxygenase-ammonia monooxygenase) pathway K10944 were removed (5 of 236 OTUs). To further validate the OTUs, centroids were compared to the October 04, 2016 NCBI-nr database using Diamond version 0.8.20 with the command blastx (Buchfink et al., 2015). The OTUs that were classified by the Last Common Ancestor algorithm from MEGAN version 6.5.8 as Eukaryote were removed (3 of 236 OTUs) (Huson and Weber, 2013). The centroid OTUs were finally classified using BLASTN (evalue cutoff of 0.02) against a custom *amo*A FunGene database described below which comprised 136 records (Fish et al., 2013). Last, the classification was added to the OTU table using a custom Perl script.

Due to poor classification results from blastx classification against the NCBI-nr database, a custom *amo*A database was created from FunGene *amo*A sequences as follows. High-quality *amo*A sequences with score above 350, size greater than 200 amino acids in length, HMM coverage of more than 85% and defined organism name were downloaded. The NCBI taxonomy of each unique record was obtained using a custom Perl script. The custom *amo*A sequences were aligned using ClustalW in MEGA7 (Kumar et al., 2016). A neighbor-joining tree (Saitou and Nei, 1987) was created to examine the phylogenetic relationships between the 138 records using as outgroup a *Nitrosococcus oceani amo*A sequence as shown in Supplementary Figure S2. Distances were computed using the Maximum Composite Likelihood method and a bootstrap test with 1000 replicates was conducted (Felsenstein, 1985). Because the *amo*A sequences clustered together at least at the Beta-proteobacteria level, the taxonomy of the records originally noted as unclassified Bacteria were updated as unclassified Beta-proteobacteria (see Supplementary Figure S2). We used the Interactive Tree of Life (iTOL) (Letunic and Bork, 2016) to visualize the tree containing the 30 most abundant *amo*A-AOB OTUs and their nearest neighbors in the custom FunGene *amo*A sequence database.

### Statistical Analyses of Gas Fluxes, Gene Abundances, and *amo*A OTUs

All statistical analyses, except Spearman correlations, were carried out in RStudio version 1.0.136 running R version 3.3.1. Generalized linear models (Bolker et al., 2009) were used to test the effect of different treatments on N_2_O fluxes and *amo*A gene copy numbers using the multcomp package (Hothorn et al., 2008) in R. The differences between treatments were analyzed within each sampling event. Treatments were considered statically significant using *p* < 0.01 as the criterion. To account for the increasing variation with the increase in the mean, we used Gamma (N_2_O emission) and Poisson family (*amo*A and 16S gene copy number) distributions as criteria to the generalized linear models. Subsequently the glht function was used to evaluate the differences among treatments (Tukey *p* ≤ 0.01). The correlation between N_2_O flux and *amo*A-AOB gene abundances were calculated by Spearman correlation analysis in Sigma Plot, version 13.0 (Systatsoftware, 2014).

The phyloseq package was used to handle the *amo*A-AOB OTU abundance data (McMurdie and Holmes, 2013). The *amo*A data were rarified to the size of the smallest sample (12,978 sequences) prior to alpha and beta diversity analyses. To determine whether AOB bacterial community diversity differed by treatment or day, Renyi indexes were calculated using the BiodiversityR package and the values for average, normally distributed Shannon and Inverse Simpson indexes were compared between treatments (Tukey’s HSD test with alpha of 0.05) using the multcomp package (Simpson, 1949; Kindt and Kindt, 2015). To test the effect of treatments on AOB bacterial community compositions, the rarified AOB data was ordinated using PCoA using the Bray distance measure. The PERMANOVA test in the vegan package was used to ascertain group significance with 9999 permutations (Oksanen et al., 2015). In parallel, the data was ordinated using correspondence analysis and group significance was assessed with between-groups analysis applying a random permutation test (999 repetitions) from the ade4 package (Dray and Dufour, 2007). Last, a permutation test for homogeneity of multivariate dispersions was run using the “betadisp” function from the vegan package. Group tests were applied for treatment (Control, N, CV+N, and V+N) and day (11, 19, and 45) groups. We also used multivariate regression tree (MRT) analyses (De’ath, 2002) in the R ‘mvpart’ package (Therneau and Atkinson, 1997; De’ath, 2007) to identify the effect of the temporal variation (time) on AOB community composition (Ouellette et al., 2012). For the MRT analysis, the rarified AOB data was log-transformed, and the tree was plotted after 500 cross-validations (Breiman et al., 1984), avoiding overfitting. Subsequently, the function rpart.pca from the mvpart package was used to plot a PCoA of the MTR.

## Results

### Weather Conditions, Greenhouse Gas Emission, and Soil Analysis

The climatic conditions during the experimental period were shown in Supplementary Figure S1A. The lowest air temperature was 7°C in the beginning of the experiment and the highest 35°C. The mean temperature during the 45-day experiment was 22°C (Supplementary Figure S1A). A similar pattern was observed in soil temperature; the temperature increased throughout the experimental period from 17 to 22°C.

Treatments with inorganic N plus vinasse application (CV or V) had higher N_2_O emission rates than treatments with only inorganic N and control. At day 11 the emission rate was low due to lack of rain during the previous period, with consumption of N_2_O in the control treatment (**Table 1**). The CO_2_ emissions were similar to N_2_O emissions, with lower emission at day 11 than in days 19 and 45. The CO_2_ emissions were higher for treatments with inorganic N plus vinasse in comparison to the control and only N (Supplementary Figure S1B).

**Table 1:** Ammonia-oxidizing bacteria (*amoA*-AOB) gene copy numbers (g^–1^ dry soil) and nitrous oxide (N_2_O) fluxes (*n* = 3) for different treatments including Control; N, inorganic N fertilizer; CV+N, concentrated vinasse plus inorganic N fertilizer; V+N, non-concentrated vinasse plus inorganic N fertilizer.

	Day 11		Day 19		Day 45	
Treatment^a^	*amo*A^b^	N_2_O-N^c^	*amo*A	N_2_O-N	*amo*A	N_2_O-N
Control	7.1 ± 2.8a	-0.07 ± 0.12a	2.8 ± 1.1a	0.11 ± 0.03a	2.4 ± 0.5a	0.24 ± 0.10a
N	12.8 ± 6.5c	0.11 ± 0.03a	738.6 ± 12.4d	0.35 ± 0.09a	41.4 ± 22.1b	8.34 ± 2.60b
CV+N	15.0 ± 6.6d	0.33 ± 0.05a	15.4 ± 8.6c	40.22 ± 7.04b	247.4 ± 146.9d	27.54 ± 14.65b
V+N	12.3 ± 5.1b	0.70 ± 0.09b	11.6 ± 3.5b	23.71 ± 7.95b	71.5 ± 14.0c	8.93 ± 1.09b

*^*a*^ Means followed by the same letter in the column at each treatment do not differ significantly by the Tukey’s test (p < 0.05); ^*b*^ ×10^*6*^ gene copies g^–*1*^ dry soil; ^*c*^ mg N m^–*2*^ d^–*1*^; Values followed by different letters are significantly different at p ≤ 0.05 using the Tukey test*.

**FIGURE 1:**
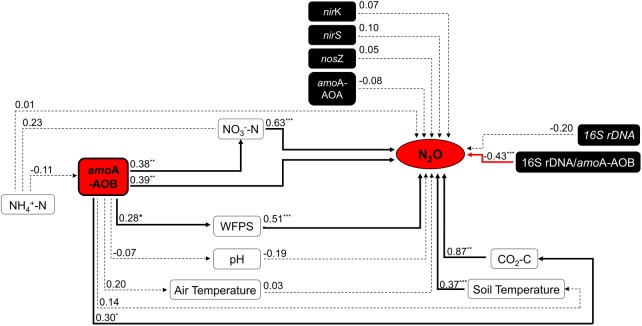
Summary of Spearman’s correlations between N_2_O emission flux (mg N m^–2^ d^–1^) and environmental variables, the abundance of bacterial *amo*A-AOB gene (gene copy g^–1^ dry soil) and the 16S rDNA*/amo*A ratio (*n* = 36). Black bold lines mean significant correlation, red bold lines significant negative correlation, and dotted lines no correlation between variables (*n* = 36). Significant difference: ^∗^*p* ≤ 0.10, ^∗∗^ *p* ≤ 0.05, and ^∗∗∗^*p* ≤ 0.01. Abbreviations: WFPS, Water-filled pore space; AOB, *amo*A belonging to ammonia-oxidizing bacteria (AOB).

Within treatments, the total NH_4_^+^-N content decreased through the time, while NO_3_^–^-N content increased (Supplementary Figures S3A,B). The soil pH had overall low variation across treatments; in the CV+N treatment the pH slightly increased while in the inorganic N and V+N treatments the pH decreased through the time (Supplementary Figure S3C). In field conditions where the spatial variation is high, the N_2_O emissions were correlated with CO_2_ emissions and other environmental parameters, including soil temperature, WFPS and NO_3_-N (*R*^2^ = 0.87; *R*^2^ = 0.37; *R*^2^ = 0.51; and *R*^2^ = 0.63, respectively) (**Figure 1**).

### Gene Abundance and AOB Community Composition Over Time

Inorganic N plus organic vinasse (CV and V) significantly increased AOB *amo*A gene copy numbers by more than 2, 8, and 51-fold at days 11, 19, and 45, respectively, compared to the control (**Table 1**). In contrast, the total bacteria (16S rDNA) gene copy number was similar for all treatments. The ratio between the abundance of the total bacteria and the *amo*A-AOB differed between treatments, and treatments with V or CV had the lowest ratio (**Table 2**). This suggested that vinasse (CV and V) plus inorganic N treatment increased the *amo*A gene copies compared with total bacteria. Furthermore, the abundance of *amo*A genes was significantly correlated with N_2_O (*R*^2^ = 0.39) and CO_2_ (*R*^2^ = 0.30) emissions; in addition, *amo*A gene abundances increased significantly with WFPS (*R*^2^ = 0.28) and soil NO_3_^–^-N (*R*^2^ = 0.38) values (**Figure 1**). However, no correlation was found between N_2_O emission and *amo*A-AOA and bacterial denitrifier (*nir*S, *nir*K, and *nos*Z) genes copy numbers (**Figure 1**).

**Table 2:** Ratios between the gene copy numbers (per gram of dry soil) of ammonia-oxidizing bacteria (*amoA*-AOB) and total bacteria 16S rDNA (*n* = 3).

	Ratio (16S rDNA/*amo*A-AOB)		
Treatments^a^	Day 11	Day 19	Day 45
Control	13891 ± 13289c	4565 ± 2029d	478 ± 169d
N	18332 ± 17408d	2974 ± 2823c	180 ± 123c
CV+N	1351 ± 847b	2005 ± 865b	89 ± 43b
V+N	998 ± 582a	1197 ± 796a	26 ± 3a

*The treatments were: Control; N, inorganic fertilizer; CV+N, mineral fertilizer plus concentrated vinasse; V+N, mineral fertilizer plus non-concentrated vinasse. ^*a*^ Values followed by the same lowercase letter in the column are not significantly different at p ≤ 0.05 using the Tukey test*.

Due to positive correlation between *amo*A-AOB gene copy numbers and N_2_O emissions, the AOB community was sequenced. A total of 1,661,482 high quality *amo*A-AOB sequences from 36 samples (4 treatments × 3 time points × 3 replicates) with an average of 46,152 reads (13,213–202,908 reads) per sample were clustered into 236 OTUs for *amo*A-AOB community analysis. Rarefaction curves indicated that the community diversity was well captured with our sequencing depth (Supplementary Figure S4).

In order to assess the effects of the treatments or day on the *amo*A-AOB community structure, the taxonomic profiles were compared at different time points using a combination of ordination techniques and dissimilarity tests. Comparative analysis of the AOB community structure revealed no clear separation at the OTU level by treatment. The PERMANOVA and correspondence analysis-between class analysis revealed no differences between treatments based on OTU-level abundances (PERMANOVA: *p* = 0.54); furthermore, the interaction between treatment and time was not significant (PERMANOVA: *p* = 0.32) (Supplementary Table S3 and Supplementary Figure S5). To further explore temporal effects, we used a multivariate regression tree (MRT) approach and PCA ordination of the by MRT analysis which further showed that the microbial community composition did not change over time (Error = 0.92) (Supplementary Figure S6). Moreover, the factors treatment or time did not affect alpha-diversity of the AOB communities (OTU richness, Chao1, Simpson, and Shannon) (Supplementary Table S4).

**FIGURE 2:**
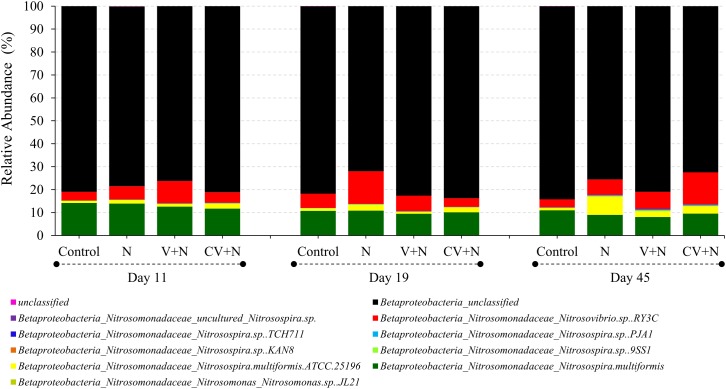
Average relative abundances of the AOB community (*n* = 36) at the level of species. The sequencing reads of *amoA*-AOB genes were assigned to their taxonomic affiliations of nitrifying bacteria that oxidize ammonia, by comparison to the *amoA* database in FunGene.

**FIGURE 3:**
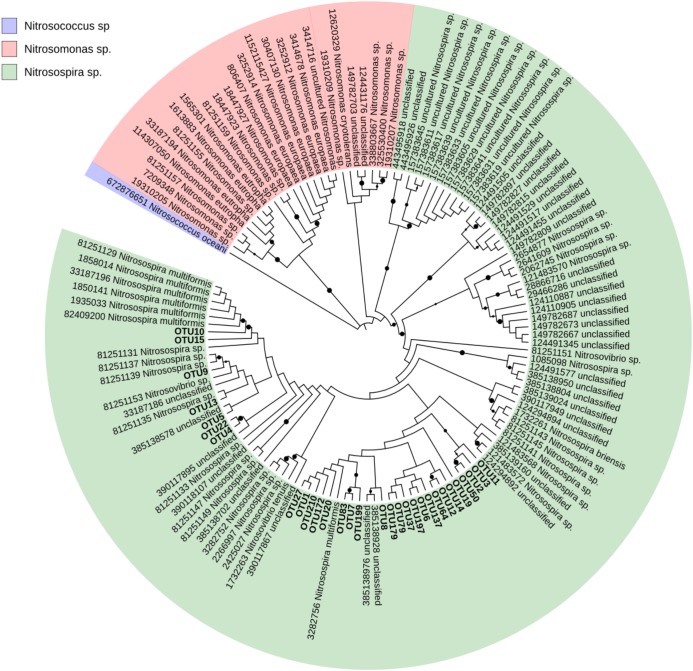
Neighbor-joining tree of the 30 most abundant sample *amo*A-AOB OTUs and their nearest neighbors in the custom FunGene *amoA* sequence database. The NCBI taxonomic classification of the database entries is included. The outgroup is *Nitrosococcus oceani*. The percentage of replicate trees in which the associated taxa clustered together in the bootstrap test (1000 replicates) are shown next to the branches (black points mean bootstrap value >75%). Evolutionary distances were computed using the Maximum Composite Likelihood method. The analysis involved 131 sequences and 305 positions and was conducted in MEGA7.

The AOB community present in the soil was composed mainly of the β-Proteobacteria phylum and the *Nitrosomonadaceae* family, of which 20.8 % belonged to the genus *Nitrosospira* and 79.2% to unclassified β-proteobacteria (**Figure 2**). Based on these results, the phylogenetic tree was constructed and all OTUs clustered with *Nitrosospira* and *Nitrosovibrio* (now included in the Nitrosospira genus) genus, except 2 OTUs (OTU 23 and OTU165) which clustered with the *Nitrosomonas* genus (**Figure 3**). *Nitrosospira* sp. PJA1 and *Nitrosovibrio* sp. RY3C were significantly positively correlated (*p* ≤ 0.10) with N_2_O-N, NO_3_-N and the number of *amo*A gene copies (**Table 3**). Surprisingly, *Nitrosospira multiformis* abundances showed significant negative correlations with N_2_O-N, NO_3_-N and the *amo*A gene copy number (*p* ≤ 0.10).

**Table 3:** Spearman’s correlation coefficients between the *amoA* OTUs (classified at the species level) and *amoA* gene copy number (determined by qPCR), N_2_O emission flux and mineral N (NH_4_^+^-N and NO_3_^–^-N) values.

Speciel-level classification^a^	N_2_O-N^b^	NO_3_^–^-N	NH_4_^+^-N	*amoA* gene copy number
*Betaproteobacteria_Nitrosomonadaceae_Nitrosomonas_Nitrosomonas* sp. JL21	–0.1	–0.02	**0.36^∗∗^**	–0.01
*Betaproteobacteria_Nitrosomonadaceae_Nitrosospira multiformis*	–**0.43^∗∗∗^**	–**0.32^∗^**	0.20	–**0.47^∗∗∗^**
*Betaproteobacteria_Nitrosomonadaceae_Nitrosospira multiformis ATCC.25196*	0.05	0.12	–0.00	**0.75^∗∗∗^**
*Betaproteobacteria_Nitrosomonadaceae_Nitrosospira* sp. 9SS1	0.19	0.25	–0.14	**0.76^∗∗∗^**
*Betaproteobacteria_Nitrosomonadaceae_Nitrosospira* sp. KAN8	0.08	0.11	–0.23	0.24
*Betaproteobacteria_Nitrosomonadaceae_Nitrosospira* sp. PJA1	**0.29^∗^**	**0.34^∗∗^**	–0.11	**0.84^∗∗∗^**
*Betaproteobacteria_Nitrosomonadaceae_Nitrosospira* sp. TCH711	0.259	0.26	–0.25	**0.66^∗∗∗^**
*Betaproteobacteria_Nitrosomonadaceae_Nitrosovibrio* sp. RY3C	**0.28^∗^**	**0.30^∗^**	–0.04	**0.81^∗∗∗^**
*Betaproteobacteria_Nitrosomonadaceae_uncultured.Nitrosospira* sp.	–0.05	–0.22	0.06	–0.26
*Betaproteobacteria_unclassified*	–0.06	–0.19	–0.16	–**0.72^∗∗∗^**
*Unclassified*	–0.22	–0.26	–0.19	–0.16

*The amoA-AOB OTUs were assigned to their taxonomic affiliations of ammonia-oxidizing bacteria (AOB) by comparison to sequences in the amoA database from FunGene. ^*a*^ Significant difference: ^∗^p ≤ 0.10, ^∗∗^p ≤ 0.05, and ^∗∗∗^p ≤ 0.01; ^*b*^ (mg N m^–2^ d^–1^). Bold values means significant correlation*.

## Discussion

Here we investigated the effect of bioenergy residues (CV and V) plus inorganic N on the abundance of bacteria and nitrifier and denitrifier genes related with N_2_O emissions, and the structure, composition and diversity of the AOB community in a tropical soil under sugarcane. Nitrification by AOB was previously determined to be the major process contributing to higher N_2_O emissions in similar tropical soil (Soares et al., 2016) planted with sugarcane due to high drainage capacity. Soares et al. (2016) showed that a NO_3_^–^ - based fertilizers had similar emission to the control treatment but neither vinasse nor straw were applied in the soil, however, the application of nitrification inhibitor together with urea decreased the N_2_O emission in 94%. The results showed that denitrification by heterotrophic microbes are not relevant in that condition. In the current study, which was carried out on the same soil type, the addition of inorganic N plus vinasse application (CV and V) boosted high N_2_O emissions. Vinasse is an organic residue rich in organic compounds with high biological oxygen demand (Fuess and Garcia, 2014). The input of labeled carbon from vinasse in the soils increases soil microbial activities including intense oxygen consumption (Renault et al., 2009) and it creates microoxic or anoxic conditions, resulting in anaerobic microsites (Torbert and Wood, 1992). Both conditions could favor denitrification, including nitrifier denitrification; however, the anaerobic conditions may prevail only for a short time, since the soil drains well and dries in a few hours. This then favors N_2_O emission by nitrification. Here, the high N_2_O emissions from treatments with inorganic N plus vinasse application (CV and V) might arise from the fertilizer N in combination with organic N and the organic carbon of vinasse.

In our study the addition of inorganic N plus vinasse application (CV and V) increased the N_2_O emissions. The high emissions were related with only AOB abundances rather than AOA or heterotrophic denitrifiers abundances. The correlation values between N_2_O and abundance of AOB is not a strong correlation, however, it is an indication that the AOB community is responsible for the N_2_O emissions. We have to emphasize that the experiment was in field conditions where the spatial variation is expected to be higher than in laboratory conditions. The AOB are considered aerobic microorganisms, which obtain energy by the oxidation of inorganic N compounds, allowing for N_2_O emissions from the soil during aerobic conditions. While nitrification by AOB is usually associated with aerobic conditions, N_2_O production by AOB is also possible via nitrification under suboxic or anoxic conditions, although these situations are still relatively unstudied in soil field conditions (Caranto et al., 2016). Furthermore, nitrifier denitrification by AOB could also play a role in N_2_O emissions in treatments with organic vinasse, under low oxygen and high concentration of nitrite (Joo et al., 2005; Spott et al., 2011; Zhao et al., 2012; Zhu et al., 2013; Bakken and Frostegård, 2017). The positive correlation between *amo*A abundance and WFPS due to rain events and vinasse application suggested that nitrification and nitrifier-denitrification processes were occurring during anaerobic conditions (Di et al., 2014). Further research is needed to explore the contribution of nitrifier nitrification versus nitrifier denitrification to N_2_O emissions under these conditions and the relevant time scales.

The application of different vinasses and inorganic N did not change the AOB community compositions, nor diversity, but did increase the abundance of AOB in the soil. Thus, it is fair to conclude that, in the short time of the experiment, the AOB community structure was resistant to the organic and inorganic fertilization, with no changes in the alpha and beta diversity. Contrasting results were found in the literature, where studies have reported changes in AOB community composition in response to N fertilizers (Glaser et al., 2010; Ouyang et al., 2016; Xiang et al., 2017). Other studies have also reported changes in AOB abundance without a corresponding change in composition with N additions (Phillips et al., 2000; He et al., 2007). Verhamme et al. (2011) found that the abundance and community structure of AOB changed only in the soil treatment with the highest ammonia concentration (200 mg N g^–1^). In our experiment, we used the N rate recommended for sugarcane fields in Brazil, which is a relatively small input rate of 0.75 mg N g^–1^. Therefore, we suggest that the community structure of AOB in soils with sugarcane was found to be unchanged after N fertilization due to the low application rate. Moreover, the AOB community in these fields may have already been adapted to the straw and annual application of inorganic fertilizer since sugarcane has been cultivated in this area for more the 20 years. Interestingly, the AOB community is composed globally of only a few species of bacteria in soils. The AOB found in soils generally belong to the β-Proteobacteria Phylum and the *Nitrosomonas* and mainly *Nitrosospira* genera (Prosser et al., 2014). There is no reported evidence of γ- Proteobacteria ammonia oxidizers (*Nitrosococcus* sp.) in soil.

Here, the AOB phylogenetic tree revealed that *Nitrosospira* was the dominant genus (99.5% of the total AOB community) in the soils under sugarcane. Recently, 16S rRNA and *amo*A gene sequencing studies have provided evidence that *Nitrosospira* spp. dominate most natural soil populations (Stephen et al., 1996; Pommerening-Röser and Koops, 2005). Surprisingly, we found only two OTUs with low abundance of *Nitrosomonas* spp. in this soil. Usually, they are prevalent in soils that have received high inputs of inorganic N (Hastings et al., 1997; Oved et al., 2001) and organic residues (Oved et al., 2001; Taylor and Bottomley, 2006; Habteselassie et al., 2013). Oved et al. (2001) and Habteselassie et al. (2013) showed that *Nitrosomonas* were not detected in soils that received inorganic fertilizer but were in soils that received liquid dairy waste and wastewater effluent. The low abundance of *Nitrosomonas* spp. in our study suggested that soil with sugarcane select for *Nitrosospira*. Even in treatments with the organic vinasse application, *Nitrosomonas* OTU abundances did not increase. On the other hand, this was the first time that vinasse was applied in the field experiment area.

The dominance of *Nitrosospira* sp. could be explained by specific conditions such as soil pH, which may have been consistent over the long period of over 20 years that this soil had been for sugarcane production. It has been postulated that pH may select for the presence of *Nitrosospira* group in acid soil (Pommerening-Röser and Koops, 2005) whereas strains of *Nitrosomonas* are not common in acidic environments (pH 4–5). The AOB isolated from acidic soils are generally *Nitrosospira* with ureolytic characteristics. For instance, some of these AOB produced urease enzymes catalyzing the breakdown of urea to ammonia (De Boer and Kowalchuk, 2001). This advantage allows the ureolytic AOB to grow at low pH with urea source (Pommerening-Röser and Koops, 2005; Ma et al., 2008). Our results showed that inorganic N application decrease soil pH over time. Therefore, the continual application of inorganic fertilizers could select the *Nitrosospira* population by lowering the soil pH.

Contrary to our hypothesis, the AOB community structure did not change with vinasse and inorganic N fertilization. The long-time inorganic N fertilization may have resulted in an AOB community that is adapted to fluctuations in mineral N in the soil, thus resulting in a diminished response of the soil AOB community structure to changes in available mineral N, affecting only the growth of the whole AOB community. Furthermore, soils with sugarcane seem to be enriched in *Nitrosospira* over *Nitrosomonas*, and the former was responsible for the N_2_O emissions from soils fertilized with organic vinasse (CV and V) and inorganic N fertilizer. This study therefore expands the information available about the microbes responsible for the N_2_O emission to define better strategies for mitigating the N_2_O emissions in sugarcane agriculture.

## Author Contributions

KL, HC, and EK designed the research. KL conducted the experiments and performed the statistical analyses. KL and AP conducted the qPCR and PCR analyses. NC performed the bioinformatic steps. KL, JvV, HC, and EK wrote the paper. All authors reviewed the manuscript.

## Conflict of Interest Statement

The authors declare that the research was conducted in the absence of any commercial or financial relationships that could be construed as a potential conflict of interest.

## Footnotes

1 https://www.ebi.ac.uk/ena/
