# Supplementary material for: Nitrosospira sp. Govern Nitrous Oxide Emissions in a Tropical Soil Amended With Residues of Bioenergy Crop
Source: Front Microbiol. 2018 Apr 10;9:674. doi: 10.3389/fmicb.2018.00674 (PMC5902487; doi:10.3389/fmicb.2018.00674)
Supplement: Supplementary file 1 [file Data_Sheet_1.docx]

Supplementary Material

*Nitrosospira sp.* govern nitrous oxide emissions in a tropical soil amended with residues of bioenergy crop

Késia Silva Lourenço^1,2,3^, Noriko A. Cassman^1,3^, Agata Pijl ^1^, Johannes A. van Veen^1,3^, Heitor Cantarella^2^, Eiko Eurya Kuramae^1*^

^1^Department of Microbial Ecology, Netherlands Institute of Ecology, Wageningen, Netherlands.

^2^Soils and Environmental Resources Center, Agronomic Institute of Campinas, Campinas, SP, Brazil.

^3^Institute of Biology Leiden, Leiden University, Leiden, Netherlands.

*** Correspondence:** Dr. Eiko Eurya Kuramae: [E.Kuramae@nioo.knaw.nl](mailto:E.Kuramae@nioo.knaw.nl)

# Supplementary Tables

**Table S1.** Chemical characteristics of the vinasse applied in the experiments.

|  |  | Concentrated vinasse - CV | Non-concentrated vinasse - V |
| --- | --- | --- | --- |
| pH |  | 4.2 | 3.9 |
| C org | (g L^-1^) | 65.3 | 31.4 |
| N tot | (g L^-1^) | 3.0 | 0.9 |
| NH_4_^+^-N | (mg L^-1^) | 100.9 | 41.6 |
| NO_3_^-^-N | (mg L^-1^) | 23.7 | 4.1 |
| P | (g kg^-1^) | 0.53 | 0.23 |
| K | (g kg^-1^) | 21.0 | 4.7 |
| C/N |  | 22/1 | 35/1 |

**Table S2.** Primers, reaction mix and thermocycler conditions used in gene abundance analysis by qPCR.

| Gene | Primers | Primer Sequence | Amplification size (bp) | Reaction | Cycling conditions | Reference |
| --- | --- | --- | --- | --- | --- | --- |
| *amoA*-AOA | Arch-amoAF  Arch-amoAR | 5’-STAATGGTCTGGCTTAGACG-3’  5’-GCGGCCATCCATCTGTATGT-3’ | 635 | 6 μL of Sybrgreen Bioline SensiFAST SYBR non-rox mix, 0.125 μL of each primer (10 pmol), 1.75 μL of BSA and 4 μL of DNA (3 ng). | 95°C-5 min.; 40x 95°C-10s, 64°C-10s, 72°C-20s | Francis *et al.* (2005) |
| *nirK* | NirK876  NirK1040 | 5'-ATYGGCGGVAYGGCGA-3'  5'-GCCTCGATCAGRTTRTGGTT-3' | 165 | 6 μL of Sybrgreen Bioline SensiFAST SYBR non-rox mix, 0.250 μL of each primer (10 pmol), 1.50 μL of BSA and 4 μL of DNA (3 ng). | 95°C-5 min.; 40x 95°C-15s, 62°C-15s, 72°C-20s | Henry *et al.* (2004) |
| *nirS* | nirScd3aF  nirSR3cd | 5'-GTSAACGTSAAGGARACSGG-3'  5'-GASTTCGGRTGSGTCTTGA-3' | 425 | 6 μL of Sybrgreen Bioline SensiFAST SYBR non-rox mix, 0.250 μL of each primer (10 pmol), 1.20 μL of BSA and 4 μL of DNA (3 ng). | 95°C-5 min.; 40x 95°C-10s, 63°C-10s, 72°C-20s | Throbäck *et al.* (2004) |
| *nosZ* | nosZ2F  nosZ2R | 5’-CGCRACGGCAASAAGGTSMSSGT-3’  5’-CAKRTGCAKSGCRTGGCAGAA-3’ | 267 | 6 μL of Sybrgreen Bioline SensiFAST SYBR non-rox mix, 0.250 μL of each primer (10 pmol), 1.20 μL of BSA and 4 μL of DNA (3 ng). | 95°C-5 min.; 40x 95°C-10s, 64°C-10s, 72°C-20s | Henry *et al.* (2006) |

**Table S3.** Result from Permutational Analysis of Variance (PERMANOVA) testing the effect of treatment or day on the ammonia-oxidizing bacterial community structure based on Bray-Curtis distance.

|  | *amoA* community | |
| --- | --- | --- |
| Main test | Pseudo-F (F) | p value |
| Treatments | 0.91 | 0.54 |
| Days | 1.41 | 0.17 |
| Treatments x Days | 1.12 | 0.32 |

**Table S4.** Alpha diversity of the ammonia-oxidizing bacterial community in treatment and timepoint comparisons. The treatments were: Control; N: inorganic N fertilizer; CV+N: concentrated vinasse plus inorganic N fertilizer; V+N: non-concentrated vinasse plus inorganic N fertilizer.

| ANOVA test ^a^ | Richness | Chao1 | | Simpson | | Shannon |  |
| --- | --- | --- | --- | --- | --- | --- | --- |
| Treatment | ns | ns | | ns | | ns |  |
| Day | ns | * | | ns | | ns |  |
| Treatment x Day | ns | ns | | ns | | ns |  |
|  | Days after vinasse application | | | | | | |
|  | 11 | | 19 | | 45 | | |
|  | *Richness* | | | | | | |
| Control | 31.00±1.73 | | 32.00±3.61 | | 30.67±4.73 | | |
| N | 35.33±2.08 | | 27.67±2.08 | | 29.67±5.86 | | |
| CV+N | 31.67±2.31 | | 31.00±0.58 | | 27.67±5.03 | | |
| V+N | 30.00±3.46 | | 28.67±1.00 | | 29.67±4.04 | | |
|  | *Chao 1* | | | | | | |
| Control | 33.30±1.31 | | 32.63±4.02 | | 34.03±4.38 | | |
| N | 37.50±3.50 | | 30.43±3.50 | | 31.17±6.71 | | |
| CV+N | 38.73±6.09 | | 38.10±8.63 | | 28.03±5.62 | | |
| V+N | 34.33±9.22 | | 30.43±1.53 | | 30.33±4.07 | | |
|  | *Simpson* | | | | | | |
| Control | 0.21±0.02 | | 0.21±0.06 | | 0.21±0.08 | | |
| N | 0.20±0.06 | | 0.18±0.08 | | 0.18±0.06 | | |
| CV+N | 0.20±0.04 | | 0.21±0.10 | | 0.19±0.04 | | |
| V+N | 0.16±0.02 | | 0.17±0.01 | | 0.15±0.03 | | |
|  | *Shannon* | | | | | | |
| Control | 2.01±0.10 | | 1.96±0.25 | | 1.96±0.14 | | |
| N | 2.06±0.23 | | 2.05±0.33 | | 2.10±0.22 | | |
| CV+N | 2.06±0.14 | | 2.01±0.29 | | 2.10±0.19 | | |
| V+N | 2.15±0.06 | | 2.11±0.03 | | 2.20±0.16 | | |

^a^ Symbols in the caption refer to overall ANOVA results for the given experiment. Significant difference: ^*^ p≤ 0.10and ns: Non-Significant.

## Supplementary Figures

**Figure S1.** Plots depicting (A) rainfall, air temperature and water-filled pore space (WFPS) and (B) daily mean fluxes of CO2-C from soils for different treatments. The treatments were: Control; N: inorganic N fertilizer; CV+N: concentrated vinasse plus inorganic N fertilizer; V+N: non-concentrated vinasse plus inorganic N fertilizer. Vertical bars indicate the standard error of the mean (n = 3).


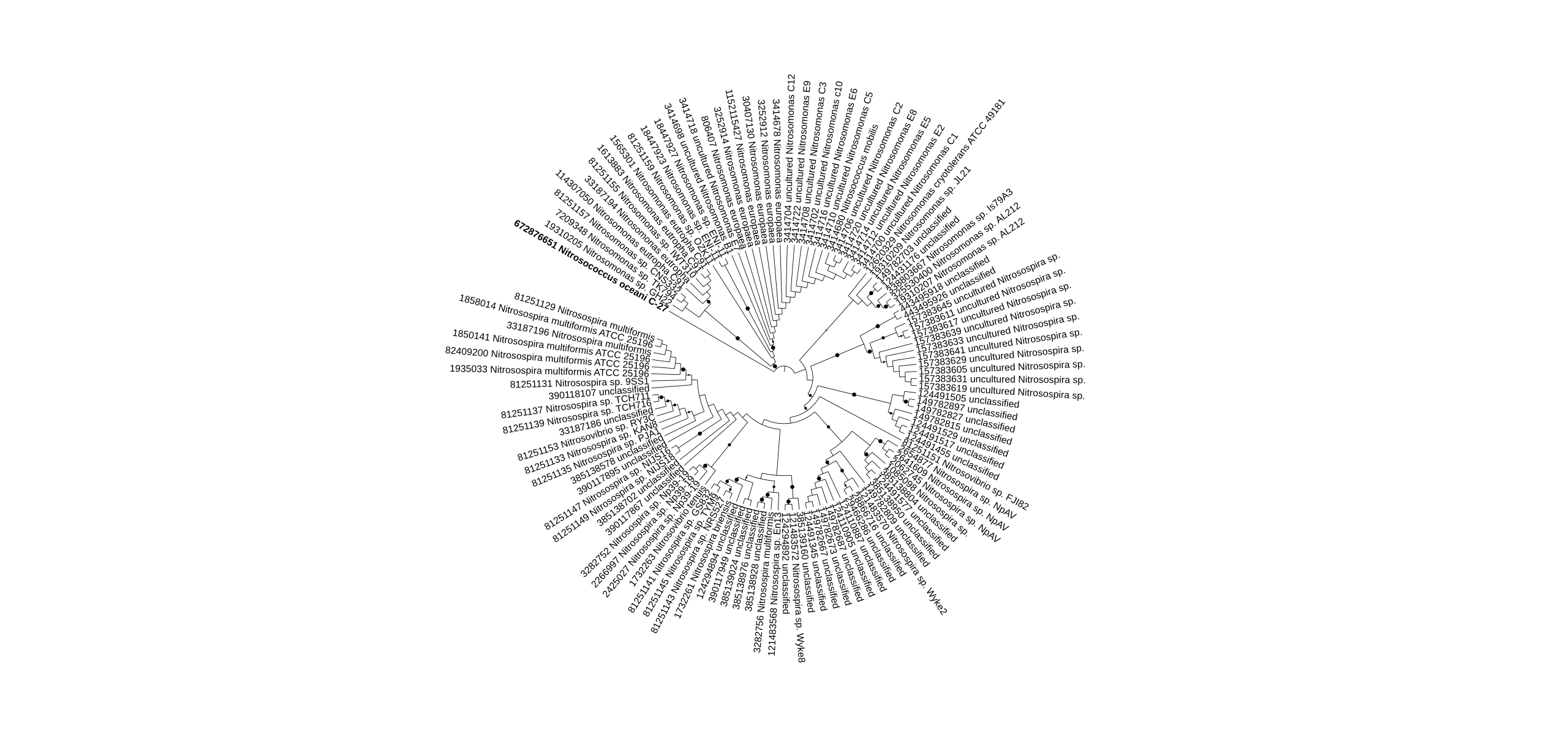


**Figure S2.** Neighbor-joining tree of the 138 *amoA* sequences taken from FunGene with NCBI taxonomy with a 25 member outgroup of Gammaproteobacterial *amoA* and the outgroup *Nitrosococcus* *oceani*. The percentage of replicate trees in which the associated taxa clustered together in the bootstrap test (1000 replicates) are shown next to the branches (bootstrap values > 75%). Evolutionary distances were computed using the Maximum Composite Likelihood method. The analysis involved 138 sequences and 461 positions and was conducted in MEGA7.

**Figure S3.** Plots of soil mineral N (NH_4_^+^-N + NO_3_^‑^-N) content (mg N kg^-1^ of dry soil) and soil pH. The treatments were: Control; N: inorganic N fertilizer; CV+N: concentrated vinasse plus inorganic N fertilizer; V+N: non-concentrated vinasse plus inorganic N fertilizer. Vertical bars indicate the standard error of the mean (n = 3). Values followed by the same lowercase letter in the column were not significantly different at p ≤ 0.05 using the Tukey test.


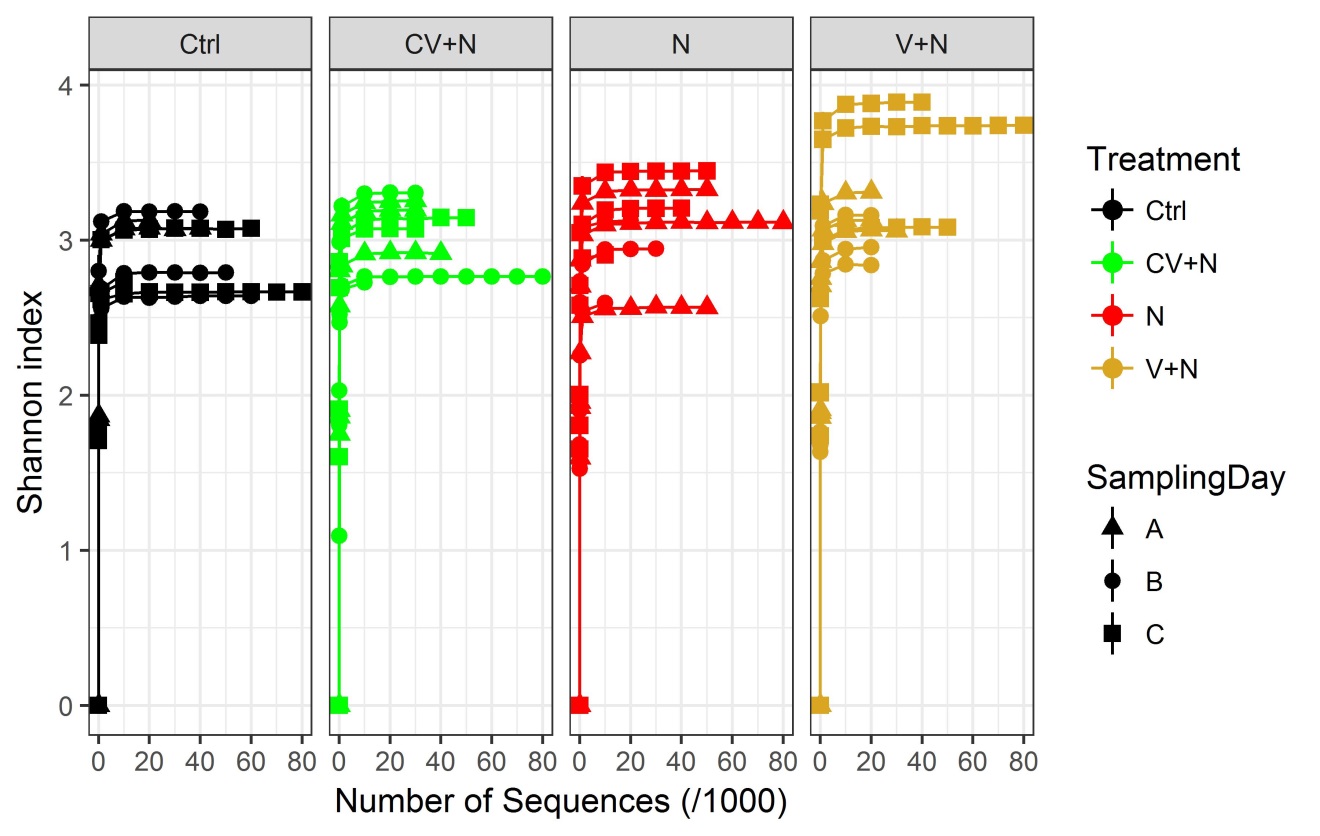


**Figure S4.** Rarefaction curves from the ammonia-oxidizing bacterial community in each treatment. The treatments were: Control; N: inorganic N fertilizer; CV+N: concentrated vinasse plus inorganic N fertilizer; V+N: non-concentrated vinasse plus inorganic N fertilizer.

1. (B)


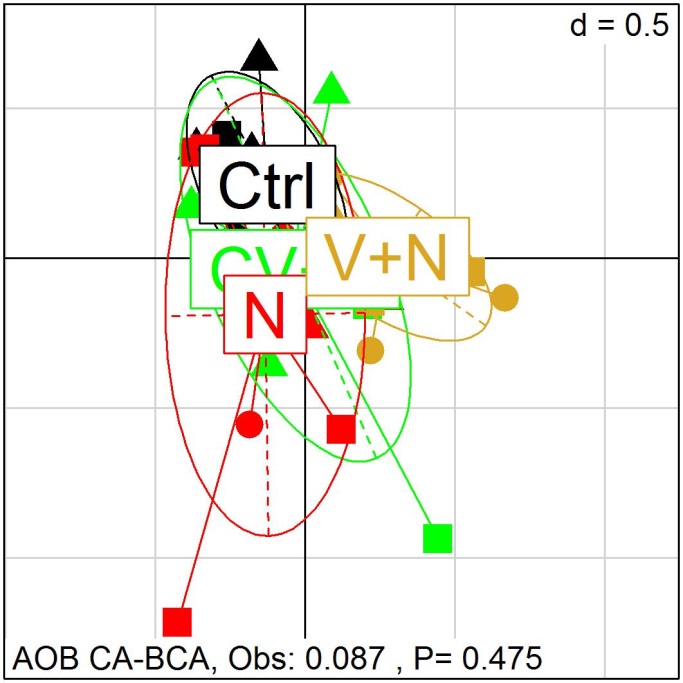

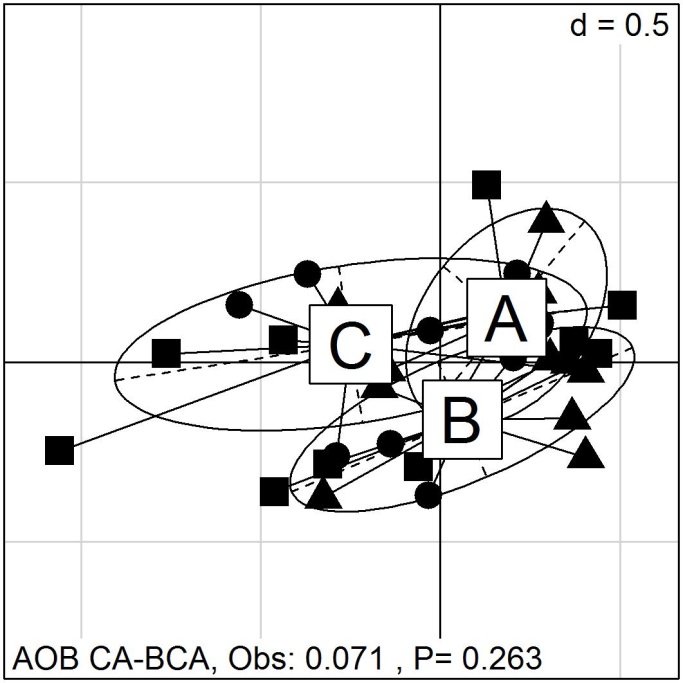


**Figure S5.** Between-Class Analysis (BCA) based on correspondence analysis of the abundance of OTUs from the ammonia-oxidizing bacteria (AOB) community (n=36) grouped by (A) treatment or (B) day. The treatments were: Control; N: inorganic N fertilizer; CV+N: concentrated vinasse plus inorganic N fertilizer; V+N: non-concentrated vinasse plus inorganic N fertilizer. The inertia between classes was 56.03% for treatment and 44.25%, for day and the Monte Carlo permutation level of significance was p=0.51 and p=0.27, respectively.

(B)

(A)

**Figure S6.** Dynamics of ammonia-oxidizing bacteria (AOB) community after vinasse plus inorganic N application. Multivariate regression tree (MRT) analysis was used to estimate the impact of time on the AOB community structure, resulting in (A) the most parsimonious tree with three different leaves (large coloured circles) defined based on AOB abundance and composition and (B) the AOB community composition within leaves represented as a PCA plot, in which small points represent individual samples and big points the mean of the samples. The grey barplot in the background indicates the OTUs whose differential abundance explains variation in the PCA plot.
